# Performance of Electronic Apex Locators, Root ZX3, NovApex N31, and Apex-S, in Irrigant Solutions of Varying Conductivity

**DOI:** 10.4317/jced.63279

**Published:** 2026-06-29

**Authors:** Lucas Rodrigues de Araújo Estrela, Luciano Tavares ngelo Cintra, Bruno Correa Azevedo, Giuliano Caixeta Serpa, Patrícia Correa Siqueira, Júlio Almeida Silva, Daniel Almeida Decurcio, Carlos Estrela

**Affiliations:** 1 DDS, MSc. Universidade Estadual Paulista – UNESP, Department of Endodontics, Araçatuba, SP, Brazil; 2 Universidade Estadual Paulista – UNESP, Department of Endodontics, Araçatuba, SP, Brazil; 3 DDS, MSc. IB Bender Postdoctoral Endodontic Program, Albert Einstein Medical Center, Philadelphia, Pennsylvania, USA; 4 DDS, MSc, PhD. Professor of Endodontics, Department of Stomatology Sciences, Federal University of Goiás, Goiânia, Brazil

## Abstract

**Background:**

The success of root canal treatment depends on precise control of the working length (WL), as it directly influences periapical healing and long-term prognosis. Optimal outcomes are achieved when obturation ends short of the radiographic apex, which requires accurate WL determination. This study evaluated the performance of electronic apex locators (EALs) in irrigant solutions with different electrical conductivities.

**Materials and Methods:**

Twenty human mandibular central incisors were assessed using Root ZX3®, NovApex N31®, and Apex-S®. Standardized reference lengths were established from the incisal edge to the root apex with a digital micrometer (DM). A size 15 K-file attached to each EAL was inserted into the canals until the devices indicated the apical foramen. The silicone stoppers were adjusted to the incisal edge, and the files were removed to measure the length with the DM. The differences between the lengths measured by the EALs and the actual WL were calculated. Statistical analysis was performed using the Friedman test followed by Tukey's post-hoc test, and the Kruskal-Wallis test with the Student-Newman-Keuls post-hoc test. The significance level was set at = 0.05.

**Results:**

Root ZX3® showed the highest accuracy (p &lt; 0.05), regardless of the irrigating solution tested. No significant differences were found between NovApex N31® and Apex-S® (p &gt; 0.05). When deionized water was used, both NovApex N31® and Apex-S® demonstrated minimal interference, whereas significant differences were observed with 2.5% NaOCl and Triton®.

**Conclusions:**

Root ZX3® demonstrated consistent accuracy across all irrigating solutions. NovApex N31® and Apex-S® performed similarly in deionized water but showed differences when compared with 2.5% NaOCl and Triton®.

## Introduction

Successful root canal treatment (RCT) depends on precise control of the working length (WL) during both canal preparation and obturation. Although often considered separate steps, these procedures are closely interrelated and directly influence periapical healing and treatment prognosis (1 - 5). Higher success rates are achieved when obturation ends just short of the radiographic apex rather than extending to it (1 - 2). However, clinical identification of the apical constriction, cementodentinal junction, and apical foramen is challenging due to anatomical variability (4 , 5). Such variations result in the apical foramen occupying different positions, most commonly central in maxillary anterior teeth (46-60%), but frequently buccally located in mandibular central incisors (44%) (6). This anatomical complexity of the root canal system continues to represent a major challenge in RCT (4 - 6). Determining the correct WL is particularly challenging, as it is influenced by anatomical variability among different tooth groups, as well as patient-related factors such as age, developmental anomalies, pathological changes, and the resources available for treatment (4 - 6). To address these challenges, various devices and techniques have been developed to improve WL determination during root canal preparation and obturation (7 , 8). Advances in technology, combined with disinfection strategies, have markedly enhanced the efficiency, accuracy, and predictability of endodontic therapy, benefiting both clinicians and patients. Electronic apex locators (EALs) have been especially valuable, reducing dependence on radiographs and minimizing the subjectivity of image interpretation (3 , 6 , 7 - 10). In clinical practice, many endodontists combine EALs with conventional radiography to improve WL determination and optimize treatment outcomes (11 , 12). Over time, numerous electronic devices have been developed and tested with different irrigating solutions to determine root canal length, using the electrical properties of periapical tissues as a reference for completing endodontic procedures (7 - 30). By analyzing these properties, the canal terminus can be located with high precision (7 , 14 - 16). Based on their operating principles, EALs have been classified into different generations (8). Among them, devices employing the ratio method, measuring impedance at two or more frequencies, have demonstrated superior reliability and accuracy (7 , 8 , 14 - 16 , 25 , 29 , 30). Clinical studies have reported promising outcomes with various EAL models (23 , 26), reinforcing their effectiveness in practice. Nevertheless, further research is warranted to evaluate new irrigant formulations (10). A comparative analysis was performed to evaluate working length (WL) measurements obtained using cone-beam computed tomography (CBCT), periapical radiographs, and an electronic apex locator (Root ZX2) in 30 teeth diagnosed with apical periodontitis (11). Initial radiographs were taken with the paralleling technique using a K-file positioned 1 mm short of the apex, and CBCT scans were acquired, followed by WL determination with the Root ZX2. The measurements obtained from CBCT (21.4 ± 2.7 mm), the apex locator (21.5 ± 3.1 mm), and radiographs (21.32 ± 3 mm) were comparable, indicating that CBCT is as accurate as the other methods for WL determination. In another study, three EALs (Root ZX2, Raypex 6, and Endo-Eze Quill) were compared in 60 teeth with asymptomatic apical periodontitis to determine the position of the K-file tip within 0 to -0.5 mm of the apical foramen (27). After extraction, the apical 4 mm of each root were trimmed to allow direct visualization of the file tip. Among the devices tested, Root ZX2 demonstrated the highest accuracy in locating the correct WL. The accuracy of EALs has been investigated under different clinical conditions (11 , 24 , 28) as well as in laboratory studies (18 , 23 , 28 , 29). In laboratory settings, the irrigating solution is the primary factor affecting the effectiveness of WL determination, rather than the pulpal or periapical status (25). In a systematic review, Tsesis et al. (25) evaluated the ability of EALs to locate the apical constriction during RCT, comparing electronic measurements with histological assessments and analyzing potential influencing factors. Their findings indicated that the accuracy of electronic WL determination depends mainly on the specific device and the type of irrigant used, while the status of the pulp tissue does not appear to affect the outcome. Advances in technology, such as the development of EALs like Root ZX3®, have provided dental professionals with greater precision and reliability. In clinical practice, irrigating substances with strong antibacterial potential have become widely available, including sodium hypochlorite (9) and Triton®-a two-component irrigant that combines the properties of sodium hypochlorite and EDTA (10). Likewise, the accuracy of electronic apex locators (EALs) has been extensively investigated in studies assessing the influence of different irrigant solutions (12 , 18 , 19 , 23 , 28 , 29). Given the recent integration of innovative technologies into endodontic practice and the limited experimental data comparing irrigants with different electrical conductivities, it is important to investigate their influence. Accordingly, this study aimed to evaluate the performance of modern electronic apex locators (EALs) in determining working length when used with irrigants of varying electrical conductivity.

## Materials and Methods

**Figure 1:**
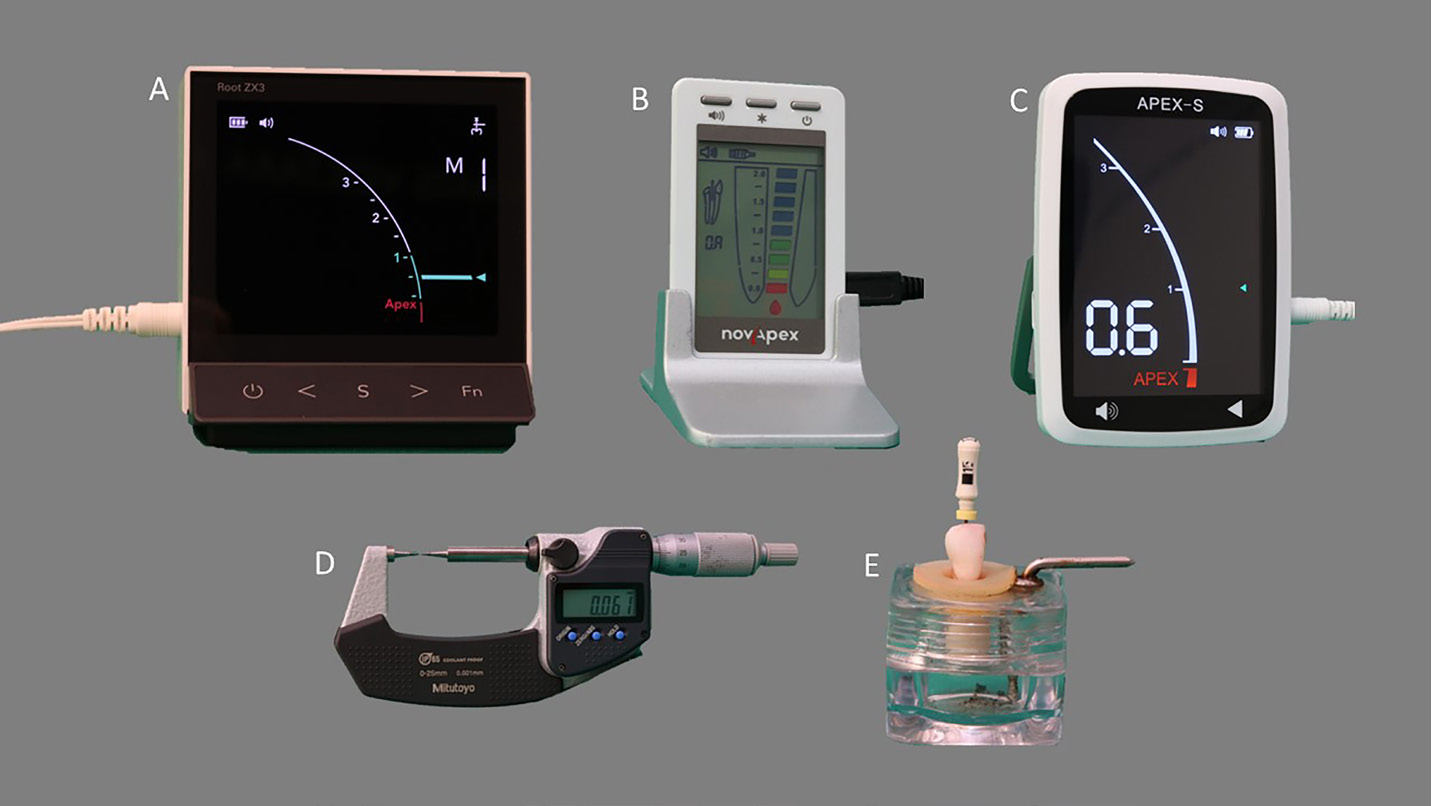
Illustrations of the EALs evaluated (A- Root ZX3®, B- NovApexN31®, and C- Apex-S®), (D) the digital micrometer, and (E) the platform simulating the use of EALs.

- Sample Selection The sample for this study consisted of human permanent mandibular incisors obtained from the Dental Urgency Department of a Public Dental School, extracted for periodontal reasons. The teeth were stored in a 0.2% thymol solution. This study was approved by the Institutional Ethics Committee of the Research Committee (Approval #82757524.4.0000.5083). Sample size calculation was performed using SigmaPlot 12.0 software (Systat Software Inc., Chicago, IL, USA). The alpha error was set at 5%, and the study power at 95%. The effect size was calculated as 0.22, indicating that a minimum of 20 samples per group was required to detect a statistically significant difference. - Inclusion and Exclusion Criteria CBCT scans were obtained after tooth extraction to verify inclusion and exclusion criteria. The study included teeth with no history of root canal treatment, orthodontic treatment, posts, crowns, internal or external root resorption, calcifications, incomplete root development, or developmental root anomalies. All selected teeth had a single, oval-shaped root canal with fully formed apices. - Imaging Methods CBCT were acquired using the following small field of view (FOV) high-resolution protocol PreXion 3D® scanner (Prexion 3d Inc, San Mateo, CA), FOV - 56 X 56 mm; acquisition voxel - 0.108 mm; exposure time - 37 seconds, tube voltage - 90 kVp, and tube current - 4 mA. The images were reconstructed using the Prexion Scanner native software and exported as multi-file DICOM images. The multifile DICOM images were reconstructed using e-Vol DX software running on a PC workstation equipped with an Intel i7-7700K processor (4.20 GHz; Intel Corp, Santa Clara, CA), an NVIDIA GeForce GTX 1070 graphics card (NVIDIA Corporation, Santa Clara, CA), a Dell P2719H monitor with a resolution of 1920 X 1080 pixels (Dell Technologies Inc, Round Rock, TX), and Windows 10 Pro (Microsoft Corp, Redmond, WA). - Sample preparation Twenty human permanent mandibular incisors were evaluated according to the following EALs used for the experiments: Root ZX3® (J. Morita, Tokyo, Japan), NovApex N31® (Foruntec, Ashkelon, Israel), and Apex-S® (Denco, Shenzhen Boson Electronic Co., Ltd, Shenzhen, China) (Fig. 1). All the teeth served as standardized references using measurements taken from the incisal edge to the root apex, with a Digital Micrometer® (0-25mm / 0.001 mm, Mitutoyo, Suzano, SP, Brazil). This device was calibrated by the Metrology Laboratory of the Brazilian Calibration Network, following the pertinent standards (ABNT NBR/ISO IEC 170/25 CAL #0031, PML 0003, certificate #07355/8). Access cavities were prepared in all teeth using a high-speed handpiece under refrigeration with round diamond burs #1011 and #2200 (FKG Dentaire, Switzerland). Root canals were explored and cleaned using stainless steel #10 and #15 K-files (Dentsply/Maillefer, Ballaigues, Switzerland) to remove debris and residual pulp tissue. The coronal third of each canal was prepared with ProTaper Next instruments (X1, #17.04, Dentsply/Maillefer, Ballaigues, Switzerland). For the WL measurement procedure, all teeth were secured in a simulation platform (Im. Brazil, São Paulo, Brazil) specifically designed for the use of EALs in prototypes or extracted teeth (Fig. 1).

1

**Figure 2:**
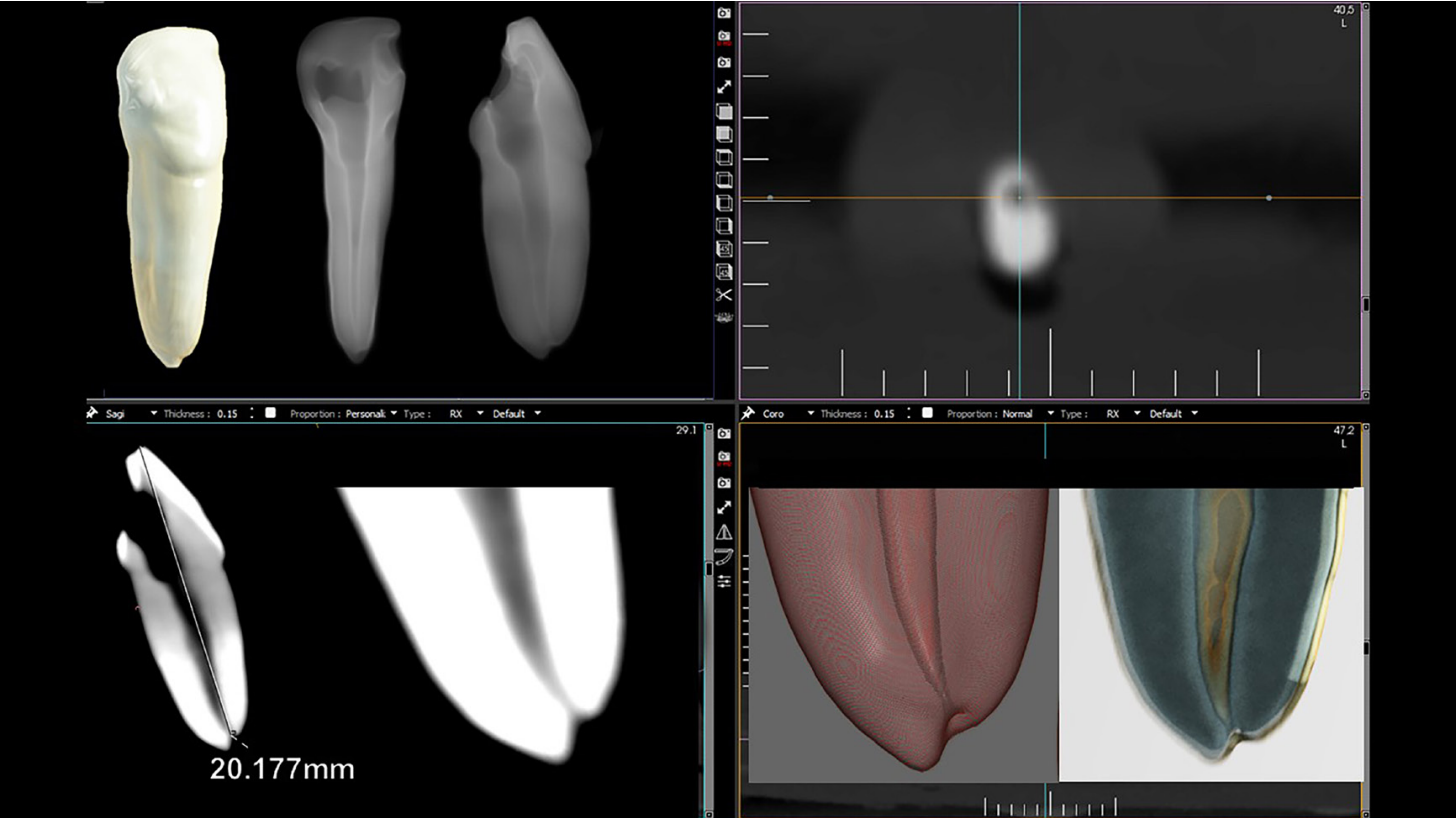
(A-D) CBCT scan with cinematic rendering of a mandibular central incisor, emphasizing the apical region, apical constriction, and apical foramen, along with measurement using the e-Vol DX software.

To mimic the periodontal ligament, the platform was filled with saline solution. During measurement, the different EALs were connected to the platform to perform odontometry. Three EALs were tested in the same twenty teeth: Root ZX3® (J. Morita, Tokyo, Japan), NovApex N31® (Foruntec, Ashkelon, Israel), and Apex-S® (Denco, Shenzhen, China). Measurements for each specimen were obtained using three irrigating solutions: deionized water® (L. 2407266/07/2024, Reymer, Goiânia, Brazil), 2.5% sodium hypochlorite (NaOCl, Asfer, São Paulo, Brazil), and Triton® (NaOCl-based solution, Brasseler, Savannah, USA). Determination of the tooth length Measurements were obtained by introducing a size 15 K-file (Maillefer, Ballaigues, Switzerland) attached to each EAL, following the operational protocols recommended by the manufacturers. Files were inserted into the canals until the devices indicated that the tip had reached the apical foramen (for 5 seconds). The silicone stoppers were then carefully adjusted to the incisal edge, and the files were removed to measure the obtained length using a digital micrometer. The differences between the measurements obtained by the three EALs and the actual tooth WL were subsequently calculated, considering the different irrigating solutions used. After each solution, the canals were thoroughly rinsed with deionized water and dried to prevent interactions between irrigants. Canal dryness was confirmed before introducing the next tested irrigant. The sequence of irrigants, deionized water®, 2.5% sodium hypochlorite, and Triton®, it was blinded to the two operators. Equal volumes of each solution were delivered using 5 mL syringes with Navitip needles, filling the entire canal. The mean of two measurements per tooth was used for statistical analysis, performed by trained operators. WL navigation and measurements were verified on CBCT scans, correlating the standardized reference measurements obtained with the digital micrometer and visualizing the apical foramen region (Fig. 2).

2

All data were recorded in an Excel spreadsheet for subsequent statistical analysis. - Statistical Analysis All statistical analyses were performed using SigmaPlot 12.0 software (Systat Software Inc., Chicago, IL, USA). Data normality was assessed using the Shapiro-Wilk test. To evaluate the effect of different irrigating solutions on EAL accuracy, the Friedman test was applied, followed by Tukey's post-hoc test. Comparisons among the different EALs were performed using the Kruskal-Wallis test, followed by the Student-Newman-Keuls post-hoc test. Differences were considered statistically significant at p &lt; 0.05. The procedural error was calculated as 0.058. Intra-examiner reliability was assessed using kappa statistics.

## Results

**Figure 3:**
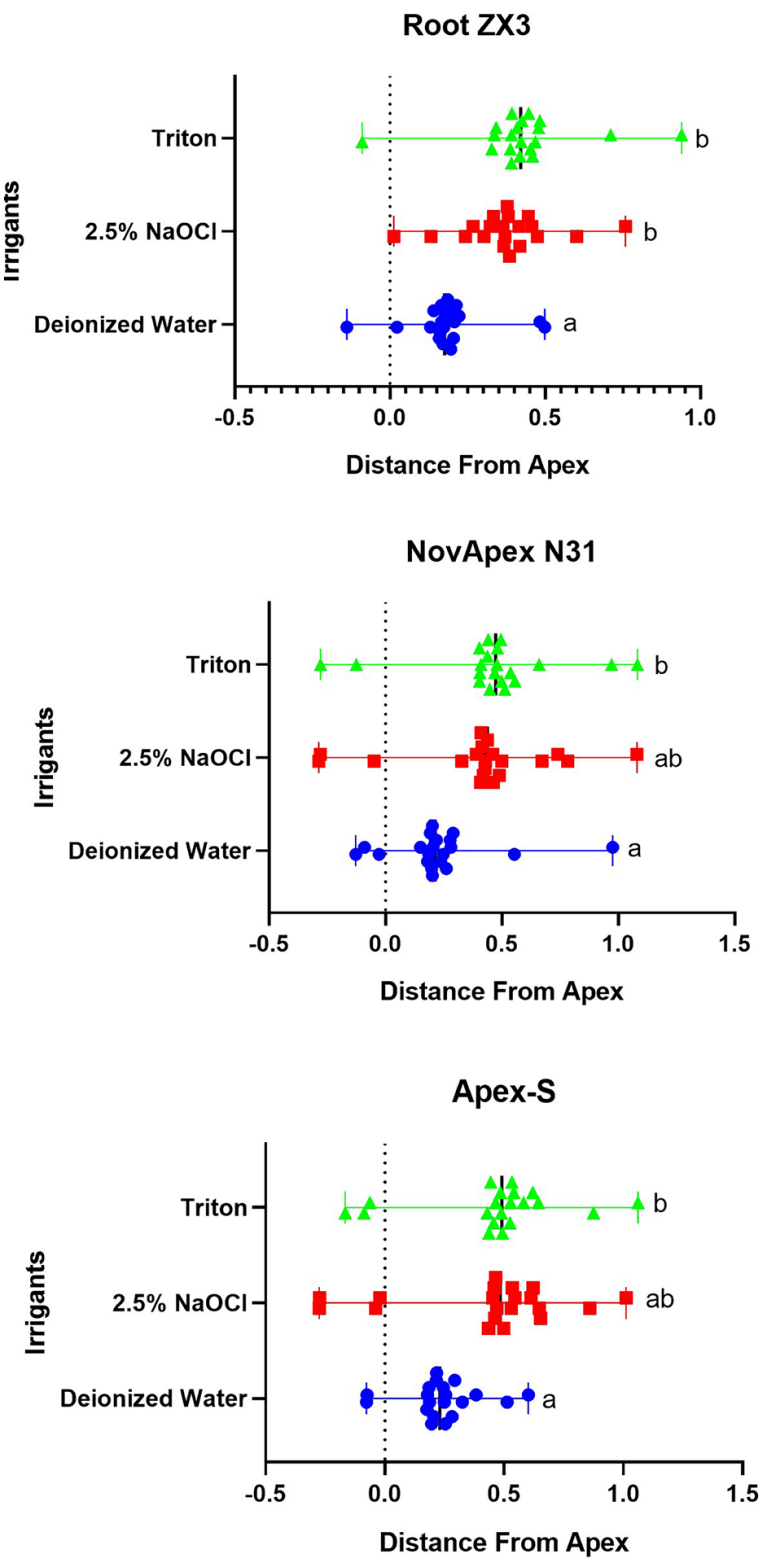
Graphics illustrating the performance of each EALs in measuring the distance to the apex across all samples, under the influence of three different irrigants (negative values show that the instrument has passed the apex).

**Figure 4:**
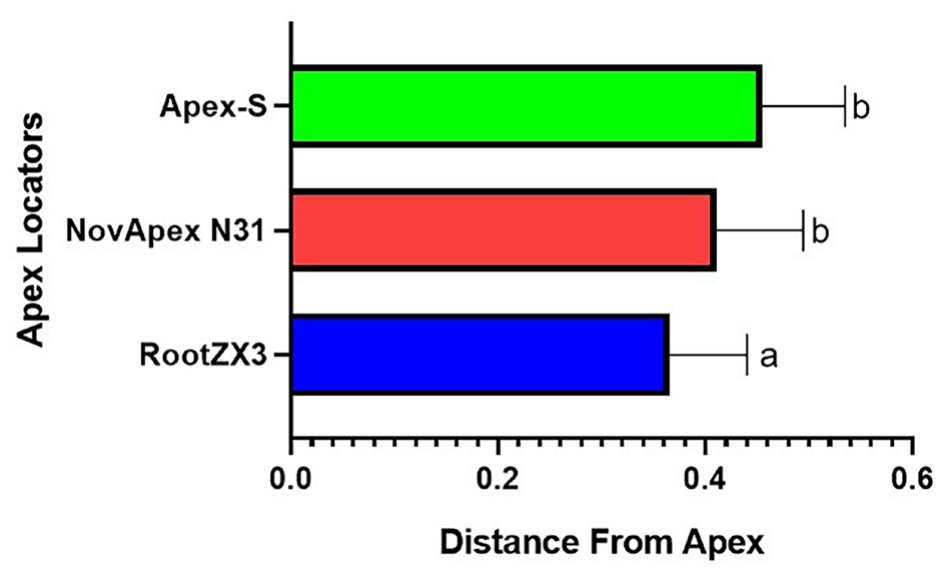
Graphic presenting the performance of EALs in measuring the distance from the apex, independent of the irrigants used.

The results are presented in Table 1 and Figures 3 and 4.

Table 1

3

4

Intra-examiner agreement was substantial, with a kappa value of 0.90. The Root ZX3® demonstrated consistent accuracy across all irrigating solutions (Figures 3 and 4). Among the tested solutions, deionized water caused the least interference in the use of the device, showing a statistically significant difference compared to 2.5% NaOCl and Triton®. Similarly, for NovApex N31® and Apex-S®, deionized water resulted in the least interference, with statistically significant differences when compared to 2.5% NaOCl and Triton® (Figs. 3,4).

## Discussion

The Root ZX3® demonstrated consistent accuracy across all three solutions, whereas the NovApex N31® and Apex-S® exhibited comparable performance, with no significant differences between them. Among the irrigating solutions tested, deionized water had the least impact on the performance of the three EALs compared with 2.5% NaOCl and Triton®. Electronic apex locators (EALs) have become essential tools in endodontic clinical practice (7 - 8 , 10 - 30). The anatomical complexity of different tooth groups, combined with the inherent limitations of periapical radiographs, two-dimensional examinations, presents significant challenges in accurately determining the ideal working length (WL) during root canal treatment (RCT) (1 - 3 , 6). More recently, the introduction of cone-beam computed tomography (CBCT), supported by advanced post-processing software, has enabled the precise identification of the apical foramen and facilitated a preliminary estimation of the ideal WL (6). When combined with the three-dimensional imaging capabilities of CBCT, the use of EALs further improves the accuracy of working length determination (11). The methodology of this study was carefully designed to evaluate newly developed electronic apex locators (EALs) and a recently introduced irrigant solution (Triton®), both of which have only recently become available in the dental market. Although deionized water is not commonly used as an endodontic irrigant, it was included in the protocol because of its chemical purity. Several key elements were incorporated into the experimental design, such as strict inclusion criteria for sample selection. A Digital Micrometer® (0-25 mm/0.001 mm), calibrated by the Metrology Laboratory, was employed for comparative measurements against the EALs. Cone-beam computed tomography (CBCT) scans, combined with post-processing software, were used to obtain pre-measurement references and to visualize images through cinematic rendering. In addition, a simulation platform specifically developed for EAL testing was employed as a prototype, using extracted teeth immersed in saline solution to simulate the periodontal ligament. Previous studies have demonstrated that different embedding media (1% agar, gelatin, alginate, saline, and floral sponge soaked in saline) show no statistically significant differences when used to assess the Root ZX electronic apex locator (EAL) in canals prepared and irrigated with 1% sodium hypochlorite (22). Based on these findings, saline solution was selected as the embedding medium in the present study. The findings of the present study showed that deionized water caused the least interference with EAL performance when compared with the irrigant solutions tested, namely 2.5% NaOCl and Triton® (an NaOCl-based solution). The influence of endodontic irrigants on the accuracy of EALs in determining working length (WL) has been extensively investigated (14 - 30). The most frequently evaluated solutions include saline (12 , 23), NaOCl (12 , 18 , 19 , 23 , 24 , 28), chlorhexidine (CHX) (12 , 18 , 24 , 28), ethylenediaminetetraacetic acid (EDTA) (18 , 28), hydrogen peroxide (18), and RC Prep (18), among others. According to a systematic review and meta-analysis by Tsesis et al. (25), early-generation EALs produced inaccurate results when exposed to conductive fluids. In contrast, devices such as the Root ZX and Justy II are capable of locating the apical foramen under various canal conditions (wet, dry, and in the presence of NaOCl) due to their multifrequency technology. Nevertheless, studies have reported that highly electroconductive irrigants, such as NaOCl, may compromise accuracy. Bilaiya et al. (28) compared the accuracy of iPex, Root ZX Mini, and Epex Pro in detecting root perforations under dry conditions and in the presence of 5% NaOCl, 2% chlorhexidine (CHX), and 17% EDTA. All three EALs detected perforations within a clinically acceptable range of 0.03-0.05 mm, with the Root ZX Mini providing the most accurate measurements in dry canals. The presence of irrigating solutions, however, influenced the accuracy of all apex locators. Similarly, Kobayashi and Suda (16) demonstrated that irrigants containing electrolytes, due to their conductive properties, can result in inaccurate readings or even prevent measurements by reducing impedance. To overcome this limitation, a multifrequency approach was developed in which the device simultaneously measures two impedances using current sources at different frequencies and calculates the ratio between the resulting electric potentials. This quotient is then displayed on the meter, indicating the file tip position within the canal. With this method, the influence of electrolytes is minimized and decreases progressively as the file approaches the apical foramen. Several studies have examined the influence of irrigant solutions on the accuracy of EALs (12 , 18 , 19 , 23 , 28 , 29). The performance of the Root ZX in the presence of various irrigants, including 2% xylocaine with 1:100,000 epinephrine, 5.25% NaOCl, RC Prep, liquid EDTA, 3% hydrogen peroxide, and 0.12% CHX, it was found to be unaffected (18). Similarly, the accuracy of different EALs (Elements Diagnostic®, Root ZX®, and Apex DSP®) was not influenced by either 0.9% saline or 1% NaOCl (23). In an in vivo study, Duran-Sindreu et al. (24) compared the performance of the iPex and Root ZX in the presence of 2.5% NaOCl and 2% CHX; the iPex demonstrated lower accuracy than the Root ZX for both solutions. Karunakar et al. (12) evaluated the effectiveness of Root ZX Mini, CanalPro CL2i, integrated apex locators, and periapical radiographs in determining WL with different irrigants (0.9% saline, 0.2% CHX, and 2.5% NaOCl), reporting no adverse effect on accuracy. Diemer et al. (29) assessed the performance of Root ZX Mini and Locapex 6 in the presence of NaOCl at varying concentrations (0.5%, 2.5%, and 5%), concluding that both EALs remained reliable regardless of concentration. Likewise, Meares and Steiman (19) investigated whether NaOCl affects the accuracy of the Root ZX. After irrigating canals with 2.125% NaOCl and taking measurements, they repeated the procedure with 5.25% NaOCl. The results showed that Root ZX accuracy was not compromised by NaOCl at either concentration. A possible explanation for the findings of the present study is that the favorable performance of deionized water, which caused the least interference with EAL accuracy compared with 2.5% NaOCl and Triton®, may be attributed to its purity. Because it contains very few dissolved ions, deionized water exhibits low conductivity, thereby allowing more accurate measurements. In contrast, NaOCl solutions contain a high concentration of dissociated ions (Na+ and Cl-), which are highly conductive and can interfere with EAL accuracy. Since electrical conductivity is directly related to the impedance measured by EALs, highly conductive solutions such as NaOCl decrease impedance, potentially reducing measurement accuracy (16 , 17). In the present study, the Root ZX3 achieved an average accuracy of 93.3%, with measurements consistently falling within -0.5 to +0.5, regardless of the irrigant used. This underscores the high precision of the device, even in the presence of NaOCl. These results align with previous studies (16 , 18 , 19), which reported that most modern apex locators are minimally affected by irrigants in the root canal. In particular, Root ZX has demonstrated superior accuracy in NaOCl environments and has proven reliable for clinical use by specialists (19). The Root ZX3, compared with its predecessor, retains the same electrical impedance technology but incorporates a more advanced system, resulting in improved measurement accuracy. According to the manufacturer (Instructions for Use, J. Morita MFG. Corp., Japan), the Root ZX3 provides faster readings and greater measurement stability. The device determines root canal length by measuring electrical impedance between the active and neutral electrodes, enabling precise apical localization. This system upgrade enhances the reliability of results during endodontic procedures, thereby improving both treatment efficiency and safety. In addition, the Root ZX3 integrates a high-frequency (HF) electrosurgical unit with indications for pulp cauterization, hemostasis, and related procedures. The NovApex (Foruntec, Ashkelon, Israel) is an EAL designed to determine WL in both dry and wet root canals. It displays the file position through color-coded segments and numerical readings, with audible signals to guide file progression. A continuous tone indicates the apical position (0.0), corresponding to the minor apical foramen. If the file extends beyond the apex, a red "Blood Drop" icon and an alert sound are activated. The file can be repositioned by gentle retraction until the icon disappears and the 0.0 reading is restored. The Apex-S® (Denco, Shenzhen, China) is a compact EAL that uses advanced technology to determine the apical position. Its high-resolution color LED screen provides clear and intuitive visual feedback, while automatic calibration ensures consistent accuracy without the need for frequent manual adjustments. Designed with user convenience in mind, it is lightweight, space-saving, and powered by a high-capacity battery, allowing extended use without frequent recharging. Although this study was conducted in vitro, the findings have clear implications for clinical endodontics. The demonstrated high accuracy of the Root ZX3® across all tested irrigating solutions suggests that clinicians can rely on this device for precise working length determination regardless of the irrigant used. The performance of the NovApex N31® and Apex-S® highlights the importance of considering irrigant type when using certain EALs, as solutions with higher electrical conductivity, such as NaOCl or Triton®, may slightly affect measurement accuracy. These results support evidence-based decision-making in selecting appropriate EALs and irrigants during root canal treatment, ultimately contributing to improved treatment efficiency, safety, and outcomes. Furthermore, the study reinforces the value of integrating advanced imaging techniques, such as CBCT, with EAL measurements to enhance precision in clinical practice. All new technologies, including EALs and novel irrigating solutions such as Triton®, must undergo rigorous testing for accuracy and reliability before being incorporated into routine dental practice. In this regard, further studies are warranted to provide additional insights that may contribute to advances in clinical endodontics. Although distilled water produces less interference with some EALs, this difference lacks clinical significance. It is important to emphasize, however, that sodium hypochlorite remains the irrigant of choice for effective microbial control in endodontic infections (9).

## Conclusions

The Root ZX3® demonstrated consistent accuracy across all irrigating solutions, whereas the NovApex N31® and Apex-S® exhibited similar performance with deionized water but showed differences when compared to 2.5% NaOCl and Triton®.

## Figures and Tables

**Table 1:** Distribution of values (mm) of the differences between the real tooth size determined by the micrometer and the tooth size determined by the EAL.

EALs	Irrigant Solutions			
	Deionized Water®(n=20)	2.5% NaOCl(n=20)	Triton®(n=20)	p
Root ZX3®	0.176 (0.160-0.207)Aa	0.374 (0.308-0.439)Ba	0.421 (0.389-0.467)Ba	<0.001
NovApex N31®	0.204 (0.182-0.274)Ab	0.428 (0.395-0.496)ABb	0.474 (0.407-0.531)Bb	0.034
Apex-S®	0.231 (0.186-0.290)Ab	0.483 (0.440-0.620)ABb	0.490 (0.438-0.572)Bb	0.025
p	0.041	0.030	0.045	

*Different letters indicate statistically significant differences (capital letters indicate differences in rows, while lower case letters in columns).

## Data Availability

The datasets used and/or analyzed during the current study are available from the corresponding author.
